# Do metastases arise from pre-existing subpopulations of cancer cells?

**DOI:** 10.1038/bjc.1983.10

**Published:** 1983-01

**Authors:** L. Weiss, J. C. Holmes, P. M. Ward

## Abstract

The hypothesis that metastases arise from pre-existing metastatic sub-populations of cancer cells with heritable metastasis-related characteristics, was tested by comparing the metastatic behaviour of cancer cells derived from pulmonary metastases with those from corresponding primary tumours, after implanting them subcutaneously in mice. In the case of KHT osteosarcomas and B16 melanomas, injected minces of metastases gave rise to more pulmonary metastases than cells derived from minces of the primary cancers generating them. However, in the case of 3LL and T241 cancers, the primary tumour minces gave rise to more pulmonary metastases than those derived from minced metastases. It is therefore concluded that the subpopulation hypothesis cannot be accepted as a general rule. When fragments of solid tumours were implanted into animals, no differences were detected between the metastatic behaviour of implants taken randomly from pulmonary metastases and the volume/age matched primary tumours generating them. These experiments thus provide no support for the hypothesis that metastases arise exclusively or predominantly from pre-existing metastatic subpopulations of cancer cells. Finally, implants of matched fragments from 3LL tumours of different volume and age, essentially produced no statistically significant differences in numbers of metastases. These observations do not therefore support the concept of a progressive evolution of subpopulations of cancer cells with heritable metastatic phenotypes during tumour growth.


*Br. J. Cancer* (1983), **47**, 081–089

### Do metastases arise from pre-existing subpopulations of cancer cells?

L. Weiss, J.C. Holmes & P.M. Ward

*Department of Experimental Pathology, Roswell Park Memorial Institute, Buffalo, New York 14263, U.S.A.*

**Summary** The hypothesis that metastases arise from pre-existing metastatic sub-populations of cancer cells with heritable metastasis-related characteristics, was tested by comparing the metastatic behaviour of cancer cells derived from pulmonary metastases with those from corresponding primary tumours, after implanting them subcutaneously in mice. In the case of KHT osteosarcomas and B16 melanomas, injected minces of metastases gave rise to more pulmonary metastases than cells derived from minces of the primary cancers generating them. However, in the case of 3LL and T241 cancers, the primary tumour minces gave rise to more pulmonary metastases than those derived from minced metastases. It is therefore concluded that the subpopulation hypothesis cannot be accepted as a general rule. When fragments of solid tumours were implanted into animals, no differences were detected between the metastatic behaviour of implants taken randomly from pulmonary metastases and the volume/age matched primary tumours generating them. These experiments thus provide no support for the hypothesis that metastases arise exclusively or predominantly from pre-existing metastatic subpopulations of cancer cells. Finally, implants of matched fragments from 3LL tumours of different volume and age, essentially produced no statistically significant differences in numbers of metastases. These observations do not therefore support the concept of a progressive evolution of subpopulations of cancer cells with heritable metastatic phenotypes during tumour growth.

It has been suggested that metastases arise exclusively or predominantly from pre-existing metastatic subpopulations of cancer cells within primary tumours, with the implication that the so-called "metastatic phenotype" is heritable. In this paper we report on some simple tests made of this interesting hypothesis.

### Materials and methods

### *Overall design of experiments*

"Source" mice were given either s.c. tumour mince injections or implants of solid tumour cylinders into their flanks. Tumours developed in these sites and metastasized into the lungs. Our basic experiment was to assay both the s.c. tumours and the metastases developing from them for (pulmonary) metastatic capability by injecting or implanting aliquots subcutaneously into the flanks of animals of "primary" or "secondary" groups, respectively.

### *Animals and tumours*

The KHT osteosarcoma (Kallman *et al.*, 1967) is carried in 6–8 week C3Hf/HeHa male mice; Lewis lung (3LL) and T241 carcinomas and the B16 (wild type) melanoma are carried in 6–8 week C57B1/6J male mice. Tumours were passaged by s.c. injections of tumour mince into the flank.

Received 27 August 1982, accepted 30 September 1982.

0007-0920/83/010081–09 $01.00

### *Tumour mince experiments*

Subcutaneous tumours and their pulmonary metastases were removed from "source" mice, minced with fine scissors, suspended in Hanks' balanced saline and 0.1 ml (18 ga. needle) injected s.c. into the flanks of mice of either "primary" or "secondary" groups respectively. Injections contained $\sim 10^5$ cancer cells, of which $\sim 30\%$ were viable on the basis of trypan blue exclusion. The size of the developing s.c. tumours was monitored by caliper measurements and, after killing animals by cervical dislocation, the volumes of the excised tumours were determined by fluid displacement. The lungs were removed and the metastases counted under a dissecting microscope ($\times 8$ magn.). All lungs removed from animals in the "primary" and "secondary" groups were monitored by examinations of stained sections throughout the experiments.

### *Tumour fragment experiments*

In an attempt to equalize the volumes of the s.c. tumours developing in the experimental groups, matched weights of s.c. and pulmonary tumours from the "source" mice were implanted into the "primary" and "secondary" groups respectively. To weight-match tumours, spherical pulmonary metastases of known diameters and primary tumour cylinders of varying lengths (obtained with 2 mm diameter cannula and trocar) were weighed for each tumour type. In the final experiments, the length of

© The Macmillan Press Ltd., 1983

s.c. tumour cylinders implanted into "primary" animals was determined by the size of pulmonary metastases implanted into "secondary" animals. In the case of KHT tumours, a mean of $1.0 \pm 0.2$ (S.E.) mg of metastases were implanted; in the 3LL tumours, $4.0 \pm 0.3$ mg, and in the T241 tumours, 3.1 $\pm 0.3$ mg. Fragments of lung from non-tumour-bearing animals were implanted into animals of the "primary" group together with tumour cylinders.

In another series of experiments made exclusively with 3LL tumours, an attempt was made to determine the effect of growth status of whole tumours on the metastatic capacity of fragments taken from them. This was accomplished by using 2 ($1 \times 2$ mm) tumour cylinders taken from "donor" cancers (of measured volumes) aged 5, 7, 9, 15, 19 and 21 days implanted s.c. into fresh recipients. In the 21 day "donor" cancers, tumour cylinders were taken from both cortical and juxtanecrotic regions and implanted into C or J sub-groups respectively. The mean cancer cell densities and mitotic indices were determined on a 5 $\mu$m thick, haemotoxylin and eosin-stained sections of representative cylinders. The recipients were killed 15 or 21 days after receiving implants, and their pulmonary metastases counted.

*Analyses of data*

The numbers of pulmonary metastases in the "primary", "secondary" and "source" experimental groups were compared by means of Student's *t*-test and the non-parametric Wilcoxon–Mann–Whitney test. Some experiments were terminated at a set time after injecting animals, and others when their s.c. tumours reached a set size as monitored by caliper measurements. Inspection of the results indicated that the average numbers of pulmonary metastases increased with some function of development-time and volume of the s.c. tumours generating them. In addition, the size of tumours developing within set times must also bear some relationship to the numbers of cancer cells injected. Although the doses of cancer cells injected s.c. into mice in the "source" and "primary" groups were reproducible as assessed by tumour growth-rate, the numbers present in the lung mince of individual "source" mice were variable as assessed by the volumes of tumours developing in recipient mice of the "secondary" groups. Therefore, in addition to comparing numbers of pulmonary metastases in *all* animals in the three experimental groups, data were also selected on the basis of development–time or volume of the s.c. tumours as indicated in Tables 1 and 4, in an attempt to minimize differences between the groups with respect to both of these variables.

### Results

*Minced tumour recipients*

The major results are summarized in Table I and their statistical analyses shown in Table II.

*KHT tumours* In both the selected and unselected series, animals in the "secondary" group, which received subcutaneous injections (s.c.i.) of minced lung metastases, developed significantly more pulmonary metastases than those in the "primary" group which received s.c.i. of minced subcutaneous tumours. The selected groups had a lower degree of significance in the *t*-tests compared with the total data (i.e., $P = 0.03$ c.f. $P = 0.002$). Over a similar period of time, no statistically significant differences were detected in the number of metastases developing in the "source" and "primary" groups.

*3LL tumours* Use of unselected data revealed no differences between the numbers of pulmonary metastases developing in the "primary" and "secondary" groups. In contrast, examination of the selected data revealed significantly more pulmonary metastases in both the "primary" and "source" than "secondary" groups.

*T241 tumour* The selected data revealed significantly more pulmonary metastases in the "primary" and "source" than in the "secondary" groups of mice. Examination of the total data revealed marginally significant differences between the "primary" and "secondary" groups, but significantly more pulmonary metastases in the "source" than in the "secondary" groups of animals.

*B16 melanoma* The unselected data revealed significantly more pulmonary metastases in the "secondary" than "primary" and "source" groups. However, this occurred against a variable metastatic background as evidenced by the presence of significantly more metastases in the "source" than in the "primary" groups. In addition, the injections in the "secondary" animals required almost twice the time to achieve tumour volumes comparable to the other 2 groups. Comparisons made between groups selected on the basis of 21–30 days elapsed-time (where both mean s.c. tumour volume and elapsed-time were not different) failed to reveal significant differences between the numbers of pulmonary metastases in the 3 groups of animals.

*Tumour fragment recipients*

The results are summarized in Table III and analyzed in Table IV.

**Table I** Numbers of lung metastases generated from s.c. injection sites in "source" animals, or following injection of cancer cell suspensions from either s.c. tumours from "source" animals ("Primary" group) or their pulmonary metastases ("Secondary" group).

| | *"Source" (S) group* | | | *"Primary" (1°) group* | | | *"Secondary" (2°) group* | | |
|---|---|---|---|---|---|---|---|---|---|
| *Tumour* | *Lung metastases Mean±s.e. (No. obs.) Median (Range)* | *S.C. Tumour Volume (ml) Mean±s.e.* | *Days Mean±s.e.* | *Lung metastases Mean±s.e. (No. obs.) Median (Range)* | *S.C. Tumour Volume (ml) Mean±s.e.* | *Days Mean±s.e.* | *Lung metastases Mean±s.e. (No. obs.) Median (Range)* | *S.C. Tumour Volume (ml) Mean±s.e.* | *Days Mean±s.e.* |
| KHT: | | | | | | | | | |
| All | 6.0±0.7(42) | 4.5±0.3 | 18±0.5 | 7.3±0.9(44) | 5.0±0.3 | 16.5±0.5 | 14.1±1.9(44) | 4.4±0.4 | 23±1.0 |
| Results | 5(0–19) | | | 6(0–23) | | | 10(0–51) | | |
| Selected | 6.8±0.8(29) | 3.8±0.3 | 20±0.3 | 6.7±1.4(18) | 4.1±0.3 | 19.5±0.5 | 13.3±2.0(31) | 3.9±0.4 | 20.5±0.5 |
| groups | 5(1–19) | | | 7(0–19) | | | 10(0–44) | | |
| Lewis Lung (3LL) | | | | | | | | | |
| All | 13.2±2.4(20) | 2.4±0.2 | 13±0.3 | 23.3±5.3(19) | 2.8±0.3 | 15±0.6 | 17.4±4.3(18) | 3.7±0.4 | 20±2.0 |
| Results | 10(2–35) | | | 12(5–94) | | | 7(5–53) | | |
| Selected | 21.0±3.1(10) | 3.2±0.2 | 14±0 | 20.3±5.5(17) | 2.9±0.3 | 15.1±0.6 | 8.0±2.5(13) | 3.2±0.4 | 15±1.2 |
| Groups | 17.5(9–35) | | | 11(5–94) | | | 6(3–37) | | |
| T241: | | | | | | | | | |
| All | 15.6±1.4(20) | 1.4±0.1 | 21±0.2 | 16.4±2.6(19) | 1.0±0.1 | 17±0.3 | 10.5±1.7(20) | 1.6±0.2 | 18±0.3 |
| Results | 15(7–34) | | | 16(0–43) | | | 9(3–28) | | |
| Selected | 15.6±1.4(19) | 1.3±0.1 | 21±0.2 | 17.9±2.6 | 1.1±0.1 | 17±0.2 | 10.9±2.0(17) | 1.3±0.2 | 18±0.4 |
| Groups | 14(7–34) | | | 16(4–43) | | | 8(3–28) | | |
| B16: | | | | | | | | | |
| All | 4.5±0.5(39) | 2.9±0.2 | 21±0.5 | 2.3±0.5(35) | 2.8±0.2 | 22±1.4 | 10.6±2.1(29) | 2.6±0.1 | 38±2.1 |
| Results | 4(0–15) | | | 1(0–8) | | | 7(0–40) | | |
| Selected | 2.3±0.7(15) | 2.9±0.4 | 25±0.4 | 2.3±0.7(18) | 3.2±0.3 | 28±1.9 | 5.3±1.6(11) | 2.5±0.2 | 27±1.1 |
| Groups | 2(0–10) | | | 1(0–8) | | | 4(0–18) | | |

The volumes of the s.c. tumours and the elapsed time from s.c. injection to death are shown for all animals, and for groups selected on the basis of volume and time as indicated.

*KHT tumours* In the unselected groups, s.c. implants derived from metastases gave rise to significantly more pulmonary metastases than implants derived from s.c. "primary" and "source" cancers. However, when the groups were selected on the basis of volume and elapsed-time, the differences between the "primary" and "secondary" groups were not statistically significant. Although the mean and median number of pulmonary metastases in the "secondary" group are approximately 3- and 5-fold that in the "primary" group, the high standard errors and wide ranges prevent a significant difference.

*3LL tumours* In the unselected groups, significantly more pulmonary metastases also occur in the "secondary" than in the "source" and "primary" groups. However, the unselected "source" and "primary" groups are also significantly different from each other. When the selection criteria are applied, the numbers of metastases in the "primary" and "secondary" groups are not significantly different.

*T241 tumours* Both in the selected and unselected groups, the numbers of metastases in the various experimental groups were not significantly different.

*Growth status of tumour implant material*

The measured volumes of 3LL tumours implanted s.c. in the flanks of C57Bl/6J mice are shown in Figure 1. After a period of 15 days in "logarithmic" growth, tumour volume remained constant up until 21 days. Apart from those animals killed at 9 days, the progressive increase in tumour volume was associated with an increase in metastasis number.

**Table II** Statistical analysis by means of Student's *t*-test and Wilcoxon-Mann-Whitney (W.M.W.) test, of the numbers of pulmonary metastases developing in the animals described in Table 1.

| | | *Source vs* 1° | | 1° *vs* 2° | | *Source vs* 2° | |
|---|---|---|---|---|---|---|---|
| *Tumour* | *Data* | *t-test* *P* | *W.M.W.* *P* | *t-test* *P* | *W.M.W.* *P* | *t-test* *P* | *W.M.W.* *P* |
| KHT | All | 0.25 | >0.2 | 0.002 | <0.01 | 0.0002 | <0.01 |
| | | | | 1°<2° | | s<2° | |
| | Selected | 0.96 | >0.2 | 0.03 | 0.05>*P*>0.01 | 0.005 | 0.05>*p*>0.01 |
| | | | | 1°<2° | | s<2° | |
| 3LL | All | 0.09 | 0.2>*P*>0.1 | 0.4 | 0.2>*P*>0.1 | 0.4 | >0.2 |
| | Selected | 0.93 | >0.2 | 0.07 | <0.01 | 0.003 | <0.01 |
| | | | | 1°>2° | | s>2° | |
| T241 | All | 0.8 | >0.2 | 0.06 | 0.1>*P*>0.05 | 0.03 | <0.01 |
| | | | | | | s>2° | |
| | Selected | 0.4 | >0.2 | 0.04 | 0.05>*P*>0.01 | 0.06 | 0.05>*P*>0.01 |
| | | | | 1°>2° | | s>2° | |
| B16 | All | 0.003 | <0.01 | 0.00006 | <0.01 | 0.002 | 0.05>*P*>0.01 |
| | | s>1° | | 1°<2° | | s<2° | |
| | Selected | 0.99 | >0.2 | 0.06 | 0.1>*P*>0.05 | 0.07 | 0.2>*P*>0.1 |

Statistically significant differences between the "source" (S), "primary" (1°) and "secondary" (2°) groups are indicated.

**Table III** Numbers of lung metastases generated from s.c. implantation sites in "source" animals, or following implantation of matched tumour fragments from either s.c. tumours from "source" animals ("primary" group) or their pulmonary metastases ("secondary" group).

| | *"Source" (S) group* | | | *"Primary" (1°) group* | | | *"Secondary" (2 ) group* | | |
|---|---|---|---|---|---|---|---|---|---|
| *Tumour* | *Lung Metastases Mean±s.e. (No. obs.) Median (Range)* | *S.C. Tumour Volume (ml) Mean±s.e.* | *Days Mean±s.e.* | *Lung Metastases Mean±s.e. (No. obs.) Median (Range)* | *S.C. Tumour Volume (ml) Mean±s.e.* | *Days Mean±s.e.* | *Lung Metastases Mean±s.e. (No. obs.) Median Range* | *S.C. Tumour Volume (ml) Mean±s.e.* | *Days Mean±s.e.* |
| KHT: | | | | | | | | | |
| All | 8.2±1.6(19) | 11.9±0.9 | 25±1.1 | 6.2±2.1(16) | 10.1±0.8 | 24±0.8 | 22.4±5.8(19) | 8.5±0.6 | 32±1.9 |
| Results | 8(0–27) | | | 2(0–28) | | | 12(0–81) | | |
| Selected | 9.2±2.7(10) | 9.9±0.9 | 26±1.6 | 8.3±3.2(10) | 10.7±1.1 | 25±0.7 | 22.5±9.2(10) | 8.3±0.8 | 27±1.1 |
| Groups | 8(0–27) | | | 3(0–28) | | | 14(0–81) | | |
| 3LL: | | | | | | | | | |
| All | 23.2±4.9(19) | 6.2±0.6 | 23±0.6 | 40.0±5.5(33) | 5.7±0.2 | 23±0.6 | 64.9±8.4(35) | 5.2±0.2 | 26±0.5 |
| Results | 19(3–96) | | | 32(2–127) | | | 55(6–257) | | |
| Selected | 19.7±3.1(9) | 5.9±0.5 | 24±0.5 | 42.7±6.4(27) | 5.6±0.2 | 24±0.6 | 59.1±6.9(31) | 5.3±0.2 | 25±0.5 |
| Groups | 20(8–36) | | | 38(2–127) | | | 55(6–191) | | |
| T241: | | | | | | | | | |
| All | 15.8±3.1(15) | 5.4±0.3 | 36±0.8 | 17.0±3.9(17) | 6.0±0.3 | 36±1.4 | 34.9±10.2(17) | 5.0±0.3 | 43±2.7 |
| Groups | 17(0–43) | | | 12(1–63) | | | 15(1–137) | | |
| Selected | 16.6±3.2(14) | 5.3±0.3 | 36±0.7 | 17.1±3.9(17) | 6.0±0.3 | 36±1.4 | 32.2±11.0(13) | 5.2±0.4 | 38±1.2 |
| Groups | 18(0–43) | | | 12(1–63) | | | 15(1–137) | | |

Volumes of s.c. tumours and the elapsed time from implantation to death are shown for all animals, and for groups selected on the basis of volume and time as indicated.

**Table IV** Statistical analyses made on numbers of pulmonary metastases in the different groups by means of Student's *t*-test and the Wilcoxon–Mann–Whitney (W.M.W.) test.

| | | "*Source*" *vs* 1° | | 1° *vs* 2° | | *Source vs* 2° | |
|---|---|---|---|---|---|---|---|
| *Tumour* | *Data* | *t-test* *P* | *W.M.W.* *P* | *t-test* *P* | *W.M.W.* *P* | *t-test* *P* | *W.M.W.* *P* |
| KHT | All | 0.45 | 0.2>*P*>0.1 | 0.02 1°<2° | 0.05>*P*>0.01 | 0.02 s<2° | 0.2>*P*>0.1 |
| | Selected | 0.83 | >0.2 | 0.16 | >0.2 | 0.18 | >0.2 |
| 3LL | All | 0.05 s<1 | 0.05>*P*>0.01 | 0.02 1°<2° | 0.05>*P*>0.01 | 0.001 s<2° | <0.01 |
| | Selected | 0.05 | 0.1>*P*>0.05 | 0.09 | 0.1>*P*>0.05 | 0.005 s<2° | <0.01 |
| T241 | All | 0.8 | >0.2 | 0.11 | >0.2 | 0.10 | >0.2 |
| | Selected | 0.9 | >0.2 | 0.16 | >0.2 | 0.17 | >0.2 |

**Figure 1** The volumes of 3LL tumours at specified times after s.c. implantation.

As shown in Table V, the relative cancer cell densities counted in sections of these tumours, approximately doubled in the 15-, 19- and 21-day specimens compared with those taken at 5, 7, and 9 days. The mitotic indices were not substantially different over this time period, although in the 21-day tumours they were twice as high in the juxtanecrotic central regions than in the cortical regions ($P=0.003$). The numbers of pulmonary metastases developing by 15 and 21 days in recipients of these tumours are also shown in Table V. The Wilcoxon–Mann–Whitney test shows that significantly more metastases were present 15 days after implantation in the recipients of 7-day tumour fragments than in those receiving 19-day tumour-fragments ($0.05>P>0.01$) and that by 21 days more metastases developed in the 19-day than 5-day recipients ($0.05>P>0.01$). Apart from these differences, neither the mean nor the median numbers of metastases developing after 15 and 21 days in all groups of recipients were significantly different.

### Discussion

Although it was suggested by Leighton in 1965 that metastases might arise from special genetically-determined subpopulations in primary cancers, serious attempts to approach the problem experimentally have only been made comparatively recently by Fidler and his colleagues (Fidler & Kripke, 1977) and subsequently by others.

Various considerations have led to the suggestion that cancer cells in metastases have different functional spectra from those in the primary cancers from which they arose. However, the validity of a number of these observations is dubious (Weiss, 1980a) and is complicated by environmental factors modifying cells (Weiss & Harlos, 1979) which may account for differences occurring after metastases have developed, as distinct from pre-existing differences in cancer cells *causing* metastases.

The issue of pre-existing metastatic subpopulations is often confused with the non-controversial issues of the heterogeneity of cancer cell populations and the establishment *in vitro* of sublines of cancer cells with different metastasis-related properties. Cancer cell heterogeneity or pleiomorphism has long been recognized as a diagnostic feature by histopathologists, and has been characterized in terms of many different experimental parameters by investigators in many different disciplines. Following the work of Fidler and his colleagues (Fidler, 1973; 1978; Kripke *et al.*, 1978) it has also been unequivocally demonstrated, albeit with a small number of different tumours, that *in vitro* cloning procedures can result in somewhat unstable (Kerbel, 1979) cell lines. When these cell lines are injected into mice, particularly by the intravenous route, they give rise to either more or less pulmonary colonies than wild-type populations from which they were derived. However, in the present context, the major issue is not whether cancer cell-lines selected and/or maintained *in vitro* exhibit different metastasis-related properties, but rather whether pre-existing metastatic sub-populations of cancer cells play a key role in the genesis of "natural" metastasis

**Table V** Effects of growth status of donor 3LL tumours on the growth and metastatic behaviour of s.c. implants in fresh recipients

| *Donor material* | | | | | | *Recipient* | | | |
|---|---|---|---|---|---|---|---|---|---|
| | | *Metastases numbers* | | | | | | *Metastases numbers* | |
| *Age of transplant (days)* | *Tumour vol (ml) (No. obs.)* | *Mean±s.e.* | *Median (Range)* | *Mean cell density ±s.e./Field* | *Mitotic Index±s.e.* | *Age (Days)* | *1° tumour Vol (ml) ±s.e.* | *Mean±s.e.* | *Median (Range)* |
| 5 | 0.04±0.01 (10) | 0.7±0.26 | 0.5 (0–2) | 72.4±1.7 (47) | 1.53±0.22 | 15 | 2.46±0.38 (10) | 3.9±0.89 | 3.5 (0–10) |
| | | | | | | 21 | 2.83±0.34 (10) | 14.8±4.83 | 13 (0–54) |
| 7 | 0.24±0.06 (10) | 3.5±0.82 | 3 (1–10) | 55.3±2.2 (48) | 1.62±0.24 | 15 | 2.22±0.32 (10) | 6.5±1.75 | 6.5 (1–20) |
| | | | | | | 21 | 3.11±0.54 (9) | 15.33±5,26 | 9 (1–49) |
| 9 | 0.48±0.06 (10) | 0.4±0.31 | 0 (0–3) | 69.9±3.7 (46) | 1.36±0.21 | 15 | 2.53±0.27 (10) | 4.7±1.63 | 3 (0–18) |
| | | | | | | 21 | 5.34±0.54 (10) | 29.8±7.51 | 23 (5–87) |
| 15 | 3.8±0.23 (10) | 12.8±3.36 | 8.5 (1–32) | 121.4±3.5 (50) | 1.56±0.17 | 15 | 2.05±0.28 (10) | 2.7±0.78 | 2 (0–8) |
| | | | | | | 21 | 4.46±0.63 (8) | 27.88±5.04 | 23.5 (9–45) |
| 19 | 5.7±0.40 (10) | 24.9±4.22 | 23 (7–45) | 125.3±2.2 (47) | 1.24±0.11 | 15 | 2.72±0.27 (9) | 2.11±0.48 | 2 (0–4) |
| | | | | | | 21 | 4.61±0.53 (8) | 29.3±9.1 | 24 (8–90) |
| 21 (C) | 5.6±0.3 (10) | 25.5±4.53 | 24.5 (6–45) | 136.9±2.5 (49) | 0.98±0.14 | 15 | 2.36±0.25 (10) | 4.4±0.98 | 3 (0–9) |
| | | | | | | 21 | 4.12±0.80 (6) | 12.67±3.84 | 11 (2–26) |
| 21 (J) | | | | 115.5±4.2 (49) | 1.97±0.29 | 15 | 2.00±0.28 (10) | 6.3±2.2 | 4 (0–21) |
| | | | | | | 21 | 3.38±0.78 (8) | 18.63±6.28 | 12.5 (5–60) |

Mean cancer cell densities were determined on 0.019 $mm^2$ microscopic fields on 5 $\mu m$ sections; mitotic indices (M.I.) are expressed in percentages of total cancer cells.

within the time-frame of the life of individual hosts. One example of many illustrating this difference is provided by N-methyl-N-nitrosourea-induced mammary carcinomas in rats, which do not spontaneously metastasize. However, metastasizing tumours have been derived from these cancers by *in vitro* and selection techniques (Williams *et al.*, 1982). If these metastasizing variants pre-exist within the tumours, we are justified in asking why the adenocarcinomas are not spontaneously metastatic within the time constraints. Mechanistic differences between B16 cell lines *in vitro* in relation to spontaneous metastasis have been identified (Weiss *et al.*, 1982).

In a review (Weiss, 1980*a*), it was suggested that a critical test of the "pre-existing metastatic subpopulation" hypothesis would be simple bioassay of the differential metastatic potential of cancer cells taken from primary cancers and their metastases in the same host. If the hypothesis is correct, it might reasonably be expected that on inoculation into similar sites in animals of the same inbred strain, cells derived from pulmonary metastases should give rise to more metastases than cells derived from the primary cancer generating them.

Experiments of this type were in fact described by Giavazzi *et al.* (1980) in which the metastasizing capacities of cancer cells from spontaneous metastases were compared with those from the transplanted murine tumours from which they arose. They noted that in general, cancer cells from a spontaneous metastasis did not show greater metastatic capacity than those from primary tumours. The authors were thus unable to support the hypothesis that metastasis are derived from selected variant cells with increased metastatic potential which pre-exist within primary tumours.

Some index of the stability in metastatic potential of the different tumour groups comes from comparison of the "source" and "primary" groups (Table II). Apart from the unselected B16 data all of the tumour systems are stable with respect to metastasis within the time-frame.

The relationships between primary tumour volume, age and growth-rate on the one hand, and metastasis on the other are extremely complex (Wood *et al.*, 1954; Weiss, 1967; Steel, 1977). However, given similar tumour types, it appears in general that the bigger the primary cancers and/or the more rapidly they grow, the greater the degree of metastases (Figure 1; Table V). In addition, *in vivo* primary tumour size within given time limits depends upon the numbers of cancer cells injected into the primary site. In the present work, it proved impossible to accurately count the numbers of viable cancer cells at the time of injection, particularly when they were obtained from the lungs of the "source" animals. This difficulty, which is avoided when cells are injected directly from cultures, is often reflected in wide ranges in numbers of metastases, mean tumour volumes and times between injection and death in animals of the unselected "primary" and "secondary" groups (Tables I–III). We therefore tend to have less confidence in differences between the unselected groups than between groups selected in such a manner as to minimize differences between subcutaneous tumour volumes and elapsed-times, even though the selection procedures reduce the numbers of animals in the individual groups.

The data given in Table 1 shows that in the case of KHT and B16 tumours, when the selected "primary" and "secondary" groups of animals are compared, cancer cells from minces of the latter gave rise to significantly more metastases than the former. However, in the case of the 3LL and T241 tumours, cells obtained from minced "primary" lesions gave rise to significantly more metastases than those derived from pulmonary metastases. Thus, if the evidence supporting the concept that metastases arise from pre-existing metastatic subpopulations in KHT and B16 tumours is accepted, we must also accept the evidence based on the same criteria that the 3LL and T241 do not. The subpopulation hypothesis cannot therefore be accepted as a general case. Finally, the experiments made with the 3LL and T241 tumours would suggest that once established, metastases from these tumours would be less likely to metastasize to additional sites within their hosts than cells from the primary cancers generating them; this phenomenon could contribute to metastatic inefficiency (Weiss, 1982).

In describing the preparation of tumour minces for injection, we noted that only $\sim$ 30% of the cancer cells obtained were viable. This raises the possibility that our results were influenced by preparative selection artifacts; whatever technique is used to isolate cells from solid tumours, this possibility remains. We therefore implanted matched undissociated fragments of solid tumours as a check on the experiments utilizing tumour minces. Unfortunately, it was not possible to implant reproducible fragments of B16 tumours, because of the ease with which they dissociated on handling.

The results with tumour fragments show that in the case of animals bearing subcutaneous tumours which were matched on the basis of volume and time after implantation, no statistically significant differences were observed in numbers of metastases in the "primary" and "secondary" groups. Thus, these experiments do not provide evidence of an heritable metastatic phenotype or that metastases arise exclusively from pre-existing metastatic subpopulations. The fact that significant differences were observed with unselected data indicates a source of potential error in the interpretation of these types of experimental data, when metastases are enumerated without reference to the volume and age of the "primary" lesions generating them.

The results given in Table V show progressive increases in both "primary" 3LL tumour volume and numbers of pulmonary metastases with time. A solitary exception was seen after 9 days, when the numbers of metastases were disproportionately low. On the one hand, this general increase in metastatic number with primary tumour volume and age would be expected if metastasis were a stochastic (random) process in terms of cancer cells. On the other hand, the increase could also have been due to the presence of increasing numbers of metastatic "mutants" or subpopulations within the implants, as their constituent cancer cell populations became larger in line with the concept of tumour progression (Foulds, 1969). In order to discriminate between these two hypotheses, similarly sized fragments from "donor" tumours of different volume and age were implanted into fresh recipients. If the older, larger tumours contained more cells of a metastatic phenotype, then it would be expected that more metastases would arise from implants of similar volume taken from them than from younger, smaller tumours. In contrast, if metastasis from these "donor" tumours were a random process, then no differences would be expected between the recipient groups. It was observed that with two exceptions, the numbers of metastases present in the fresh recipients after 15 or 21 days were not significantly different. The two exceptions were revealed by Wilcoxon–Mann–Whitney tests but not *t*-tests, between 19- and 7-day implants where significantly ($0.05 > P > 0.01$) more metastases were found in the 7-day recipients after 15 days, and between 19- and 5-day implants after 21 days, significantly ($0.05 > P > 0.01$) more metastases were found in the 19-day tumour recipients.

Potential differences between the numbers of metastases in the different groups were not obscured by either different relative cancer cell densities or different mitotic indices in the implanted tumour fragments. The higher relative densities in the 15-, 19- and 21-day implants coupled with a high frequency of metastatic phenotypes should have caused significantly higher numbers of metastases than in the 5-, 7- and 9-day tumour recipients. This was not observed. The mitotic indices in the groups of "donor" tumours were also not significantly different, with the exception of samples taken from the juxtanecrotic regions of the 21-day "donors". Thus, while these experiments are compatible with the concept of a random process in metastasis at cancer cell level, they offer no support to the metastatic "subpopulation" hypothesis.

In spite of a plethora of papers, there is little or no direct evidence to support the hypothesis that spontaneous metastases arise exclusively or predominantly from pre-existing metastatic subpopulations in the cancer generating them, which consist of cancer cells with *heritable* (stable) metastatic phenotypes. A corollary of this hypothesis would be that the major cell populations in metastases consists of cells of a metastatic phenotype, whereas these would compose only a comparatively minor subpopulation within the original primary tumour. In contrast, it has been proposed (Weiss, 1980*b*) that cells entering the metastatic process do so from "transient metastatic compartments." and that after allowance is made for pathophysiologic differences between primary and metastatic lesions, metastases are no more likely to metastasize than their parent primary tumour. The presnt studies support this proposal. The issue is of considerable clinical importance since metastasis of metastases is a key feature of the natural history of cancer in Man (Sugarbaker *et al.*, 1971); many tumours first metastasize to "generalizing" sites (e.g. lymphnodes, liver and lungs), from which tertiary and subsequent metastases arise (Bross & Blumenson, 1976).

Our thanks are due to Mr. D. Graham for his skilled technical support. We also thank Dr. R. Hill, Ontario Cancer Institute, for providing us with the initial KHT tumours.

This work was supported in part by Grant CD-21 from the American Cancer Society, Inc.

### References

BROSS, I.D.J & BLUMENSON, L.E. (1976). Metastatic sites that produce generalized cancer identification and kinetics of generalizing sites. In *Fundamental Aspects of Metastasis* (Ed. Weiss),. New York: American Elsevier, p. 359.

FIDLER, I.J. (1973). Selection of successive tumor lines for metastasis. *Nature* (New Biol). **242.** 148.

FIDLER, I.J. (1978). Tumour heterogeneity and the biology of cancer invasion and metastasis. *Cancer Res.*, **38,** 2651.

FIDLER, I.J. & KRIPKE, M.L. (1977). Metastasis results from pre-existing variant cells within a malignant tumor. *Science*, **197,**893.

FOULDS, L. (1969). *Neoplastic Development,* Vol. 1. London. Academic Press: p. 69.

GIAVAZZI, R., ALLESSANDRI, G., SPREAFICO, F., GARATTINI, S. & MANTOVANI, A. (1980). Metastasizing capacity of tumour spontaneous metastases of transplanted murine tumours. *Br. J. Cancer*, **42**, 462.

KALLMAN, R.F., SILINI, G. & VAN PUTTEN, L.M. (1967). Factors influencing the quantitative estimation of the *in vivo* survival of cells from solid tumours. *J. Nat. Cancer Inst.*, **39**, 539.

KERBEL, R.S. (1979). Immunologic studies of membrane mutants of a highly metastatic murine tumor. *Am. J. Pathol.*, **97**, 609.

KRIPKE, M.L., GRUYS, E. & FIDLER, I.J. (1978). Metastatic heterogeneity of cells from an ultraviolet light-induced murine fibrosarcoma of recent origin. *Cancer Res.*, **38**, 1962.

LEIGHTON, J. (1965). Inherant malignancy of cancer cells possibly limited by genetically differing cells in the same tumour. *Acta Cytol.*, **9**, 138.

STEEL, G.G. (1977). *Growth Kinetics of Tumours*. Oxford, Clarendon Press: pp. 5–55.

SUGARBAKER, E.V., COHEN, A.M. & KETCHAM, A.S. (1971). Do Metastases Metastasize? *Ann. Surg.*, **174**, 161.

WEISS, L. (1967). *The Cells Periphery, Metastasis and Other Contact Phenomena*: Amsterdam, North Holland Publ. Co. p. 311.

WEISS, L. (1980a). Differences between cancer cells in primary and secondary tumors. *Pathobiol. Ann.*, **10**, 51.

WEISS, L. (1980b). The cell periphery and metastasis. In *Brain Metastasis* (Ed. Weiss *et al*): Boston, G.K. Hall, p. 30.

WEISS, L. (1982). Metastatic inefficiency. In: *Liver Metastasis* (Eds. Weiss & Gilbert) Boston: G.K. Hall, p. 126.

WEISS, L. & HARLOS, J.P. (1979). Differences in the peripheries of Walker cancer cells growing in different sites in the rat. *Cancer Res.*, **39**, 2481.

WEISS, L., MAYHEW, E., RAPP, D.G. & HOLMES, J.C. (1982). Metastatic inefficiency in mice bearing B16 Melanomas. *Br. J. Cancer*, **45**, 44.

WILLIAMS, J.C., GUSTERSON, B.A. & COOMBES, R.C. (1982). Spontaneously metastasizing variants derived from MNU-induced rat mammary tumour. *Br. J. Cancer*, **45**, 588.

WOOD, J.S., HOLYOKE, E.D., CLASON, W.P.C., SOMMERS, S.C. & WARREN, S. (1954). An experimental study of the relationship between tumor size and number of lung metastases. *Cancer*, **7**, 437.


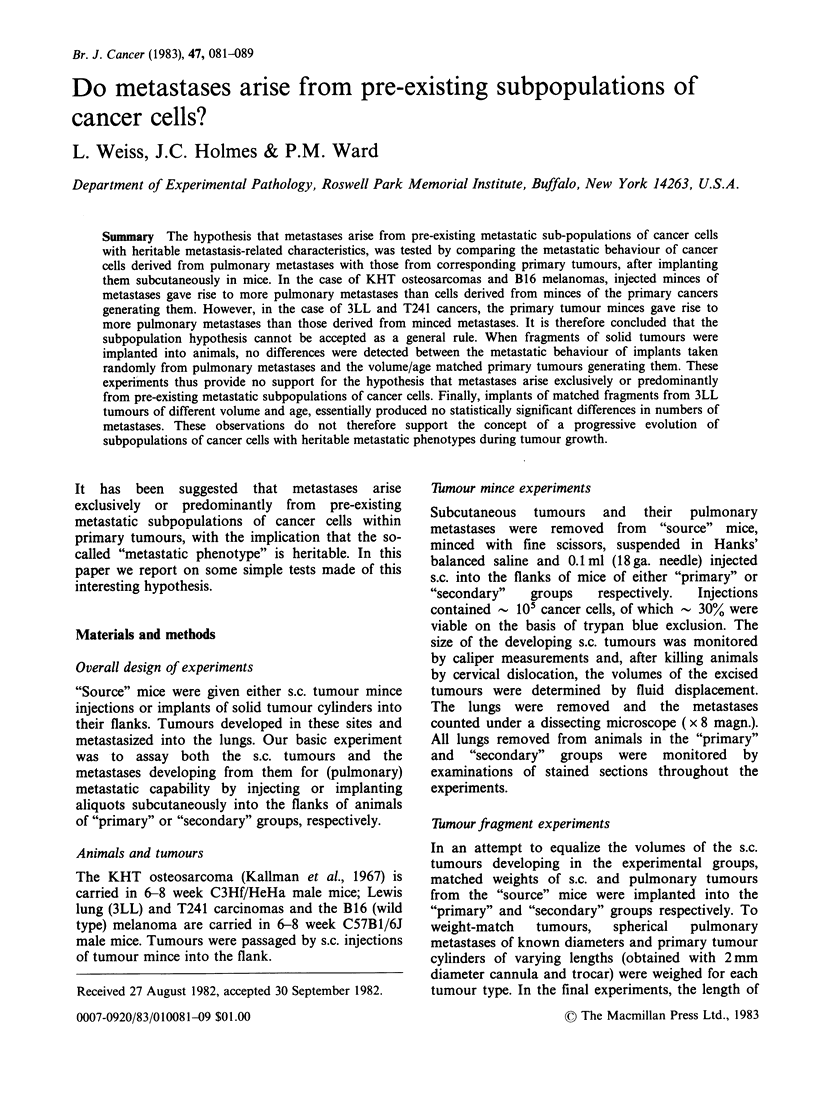

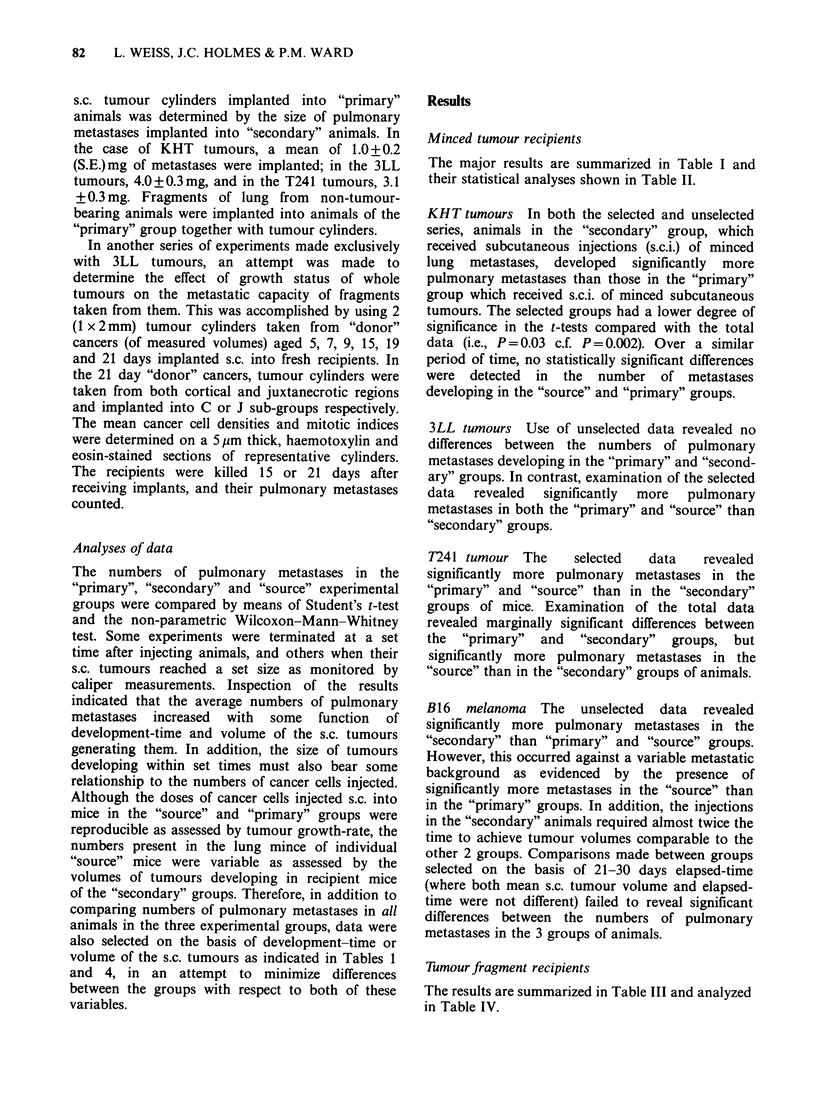

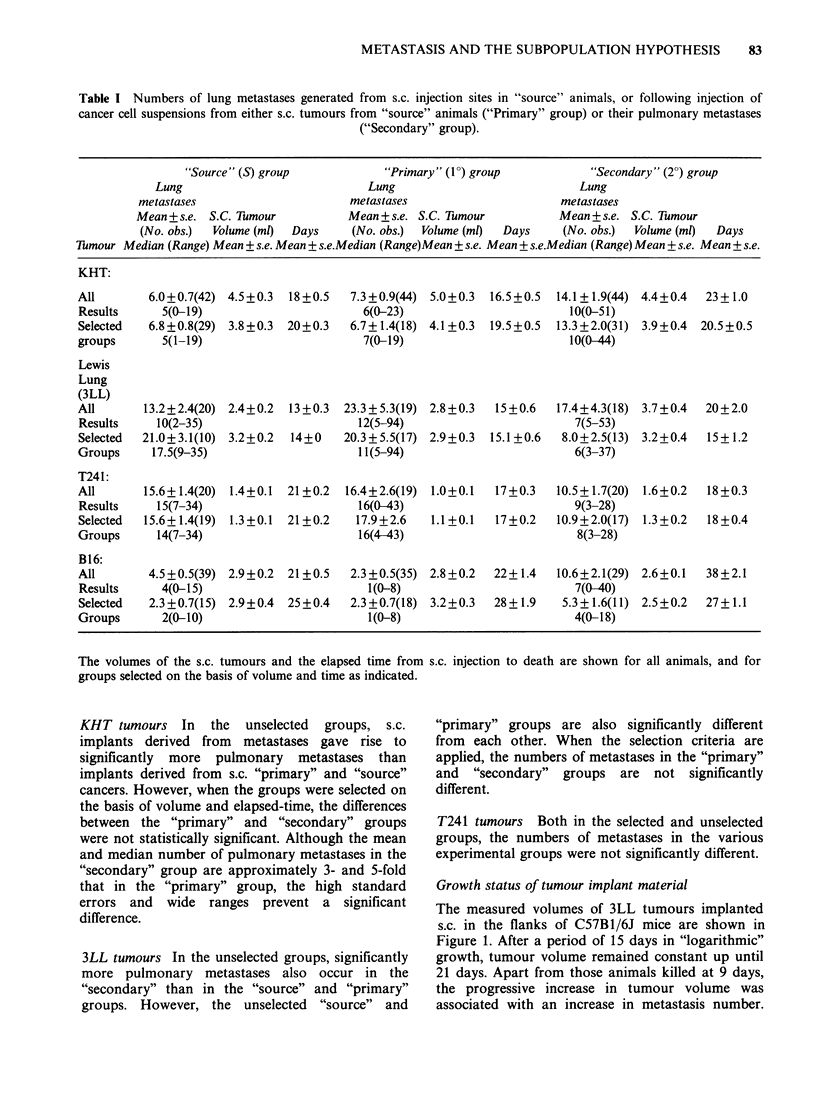

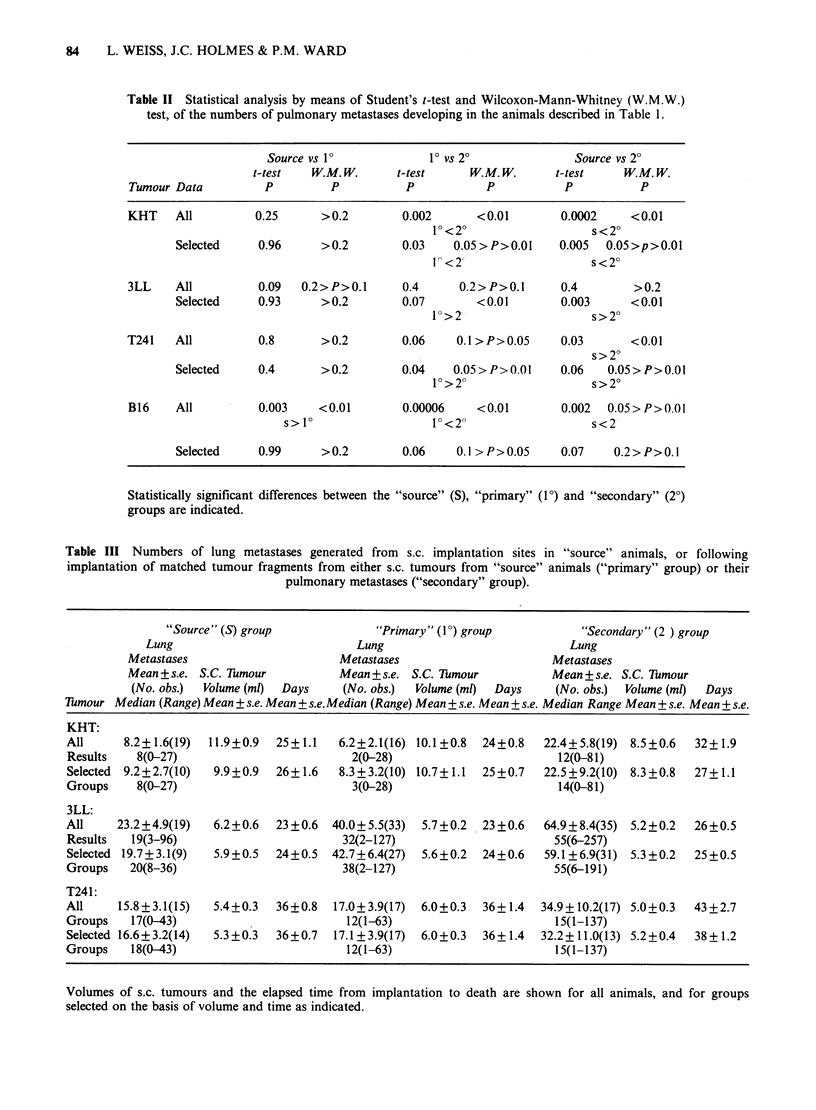

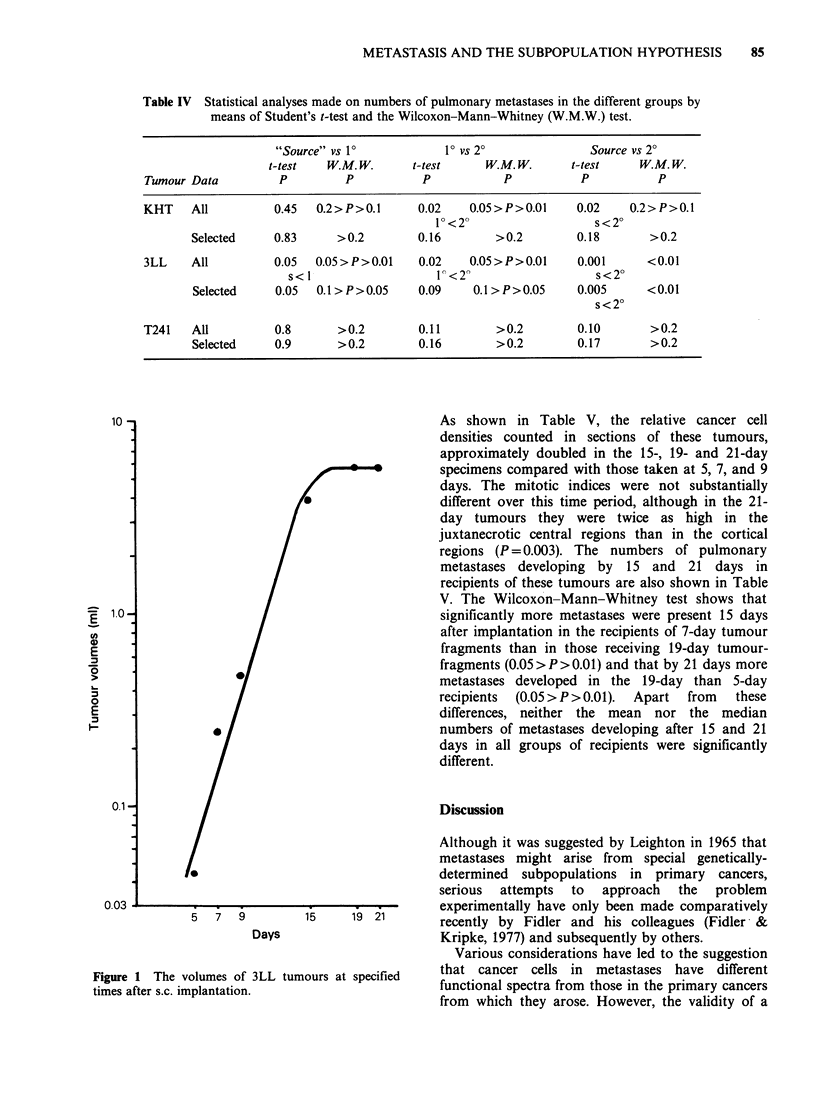

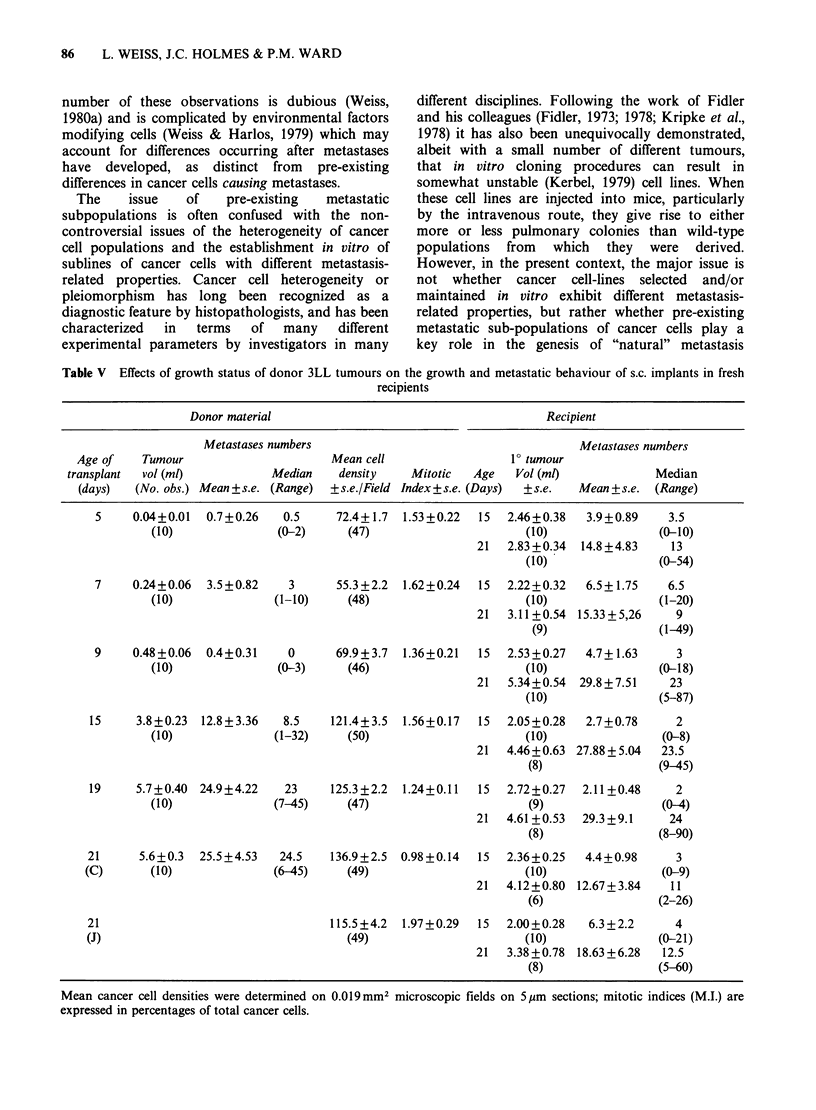

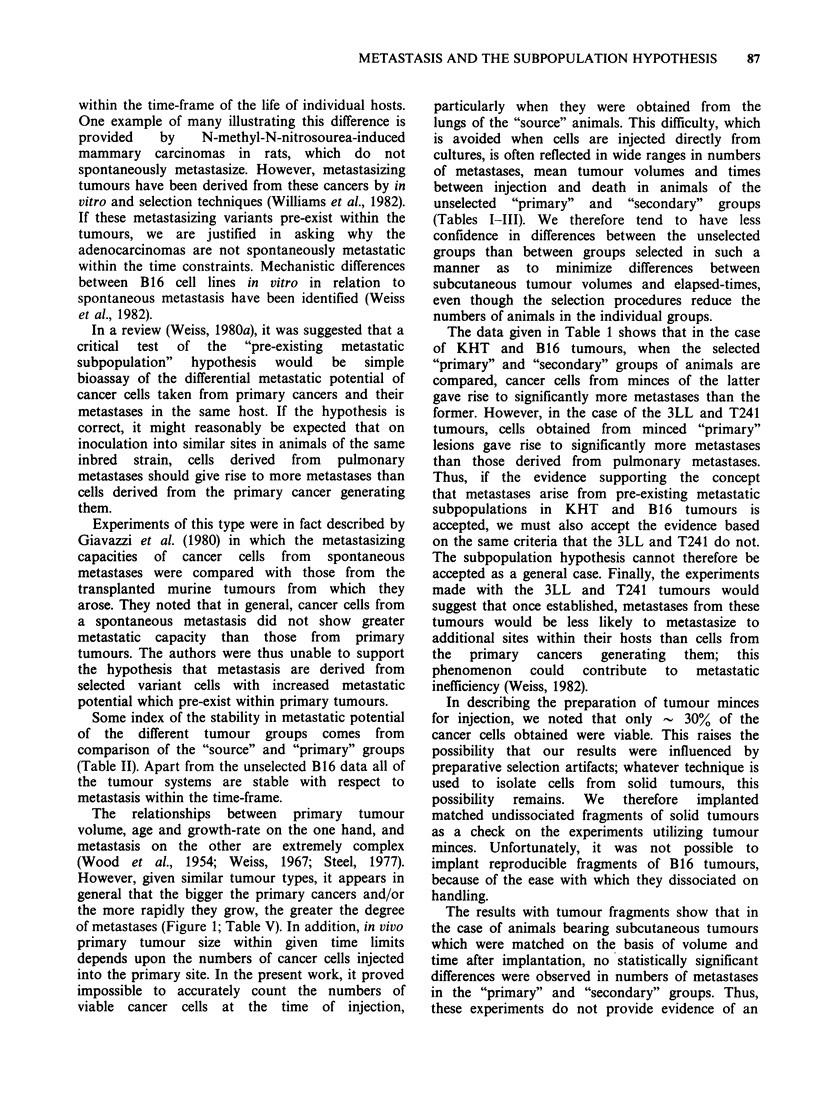

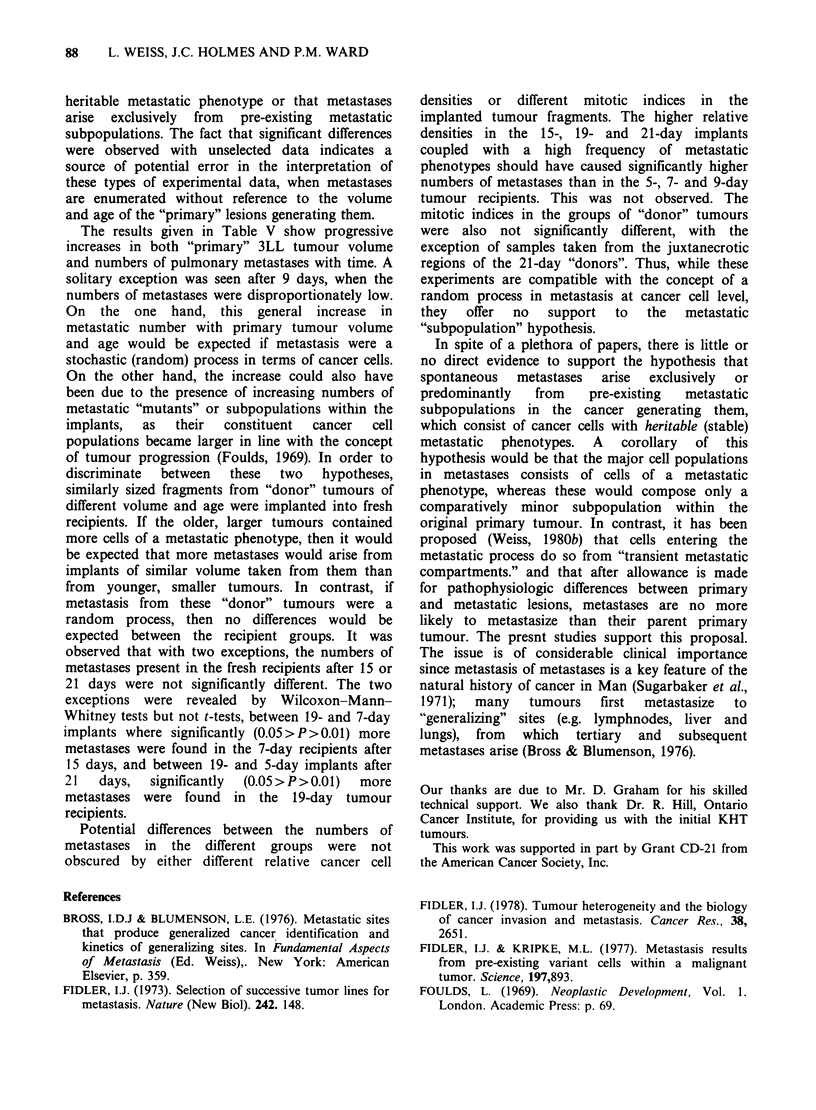

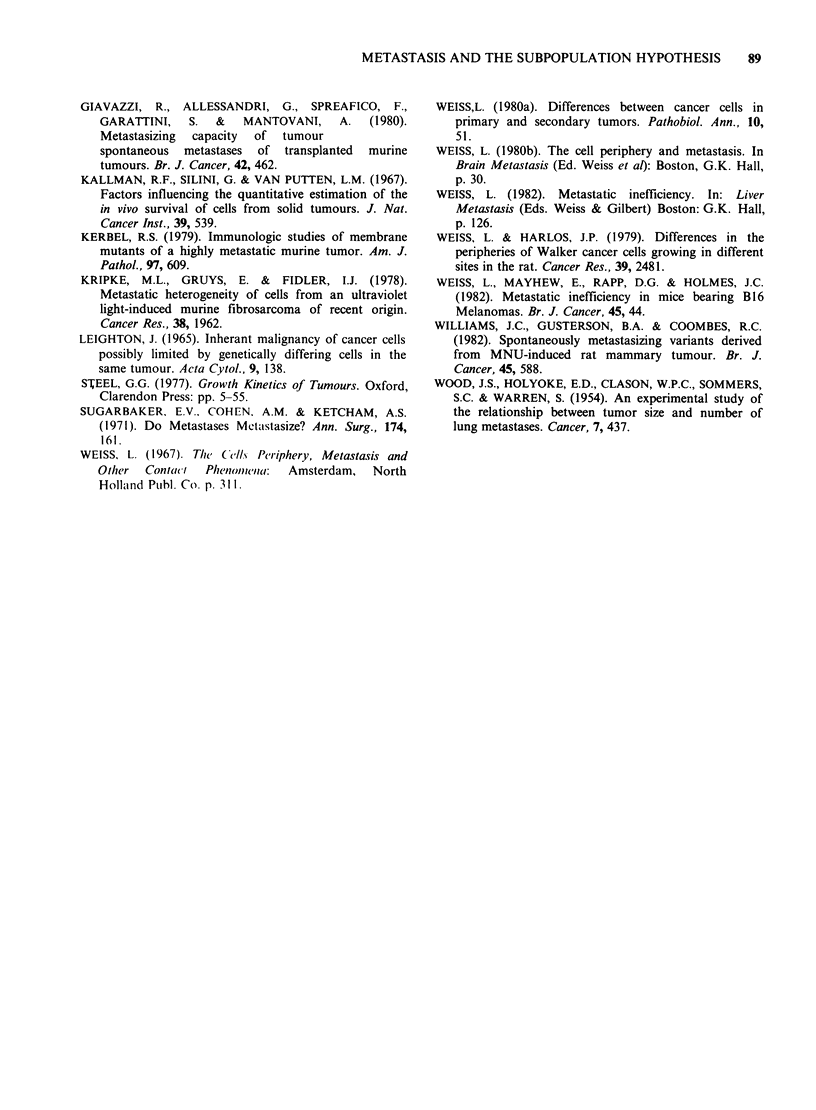


## References

[OCR_01102] Fidler I. J., Kripke M. L. (1977). Metastasis results from preexisting variant cells within a malignant tumor.. Science.

[OCR_01093] Fidler I. J. (1973). Selection of successive tumour lines for metastasis.. Nat New Biol.

[OCR_01097] Fidler I. J. (1978). Tumor heterogeneity and the biology of cancer invasion and metastasis.. Cancer Res.

[OCR_01113] Giavazzi R., Alessandri G., Spreafico F., Garattini S., Mantovani A. (1980). Metastasizing capacity of tumour cells from spontaneous metastases of transplanted murine tumours.. Br J Cancer.

[OCR_01121] Kallman R. F., Silini G., Van Putten L. M. (1967). Factors influencing the quantitative estimation of the in vivo survival of cells from solid tumors.. J Natl Cancer Inst.

[OCR_01127] Kerbel R. S. (1979). Immunologic studies of membrane mutants of a highly metastatic murine tumor.. Am J Pathol.

[OCR_01147] Sugarbaker E. V., Cohen A. M., Ketcham A. S. (1971). Do metastases metastasize?. Ann Surg.

[OCR_01188] WOOD J. S., HOLYOKE E. D., CLASON W. P., SOMMERS S. C., WARREN S. (1954). An experimental study of the relationship between tumor size and number of lung metastases.. Cancer.

[OCR_01172] Weiss L., Harlos J. P. (1979). Differences in the peripheries of Walker Cancer cells growing in different sites in the rat.. Cancer Res.

[OCR_01177] Weiss L., Mayhew E., Rapp D. G., Holmes J. C. (1982). Metastatic inefficiency in mice bearing B16 melanomas.. Br J Cancer.

[OCR_01157] Weiss L. (1980). Metastasis: differences between cancer cells in primary and secondary tumors.. Pathobiol Annu.

[OCR_01182] Williams J. C., Gusterson B. A., Coombes R. C. (1982). Spontaneously metastasizing variants derived from MNU-induced rat mammary tumour.. Br J Cancer.

